# Bleeding in a 43 Year Old Female: A Rare Disease

**DOI:** 10.1155/2013/159309

**Published:** 2013-03-30

**Authors:** Nwabundo Nwankwo, Olayinka Akinola, Aibek E. Mirrakhimov, Nkemakolam A. Iroegbu

**Affiliations:** Department of Internal Medicine, Saint Joseph Hospital, 2900 N. Lake Shore Drive, Chicago, IL 60657, USA

## Abstract

A 43-year-old gravida 2 para 2 Caucasian female with a past medical history of menorrhagia secondary to uterine fibroids and thyroid disease presented to the emergency department with complaints of bruising in her oral mucosa and vaginal bleeding. One week prior to this presentation, she was transfused with two units of packed red blood cells because of symptomatic anemia secondary to menorrhagia. Physical examination was normal, except for petechiae on the abdomen and the lower extremities as well as purpuric lesions on the buccal mucosa. Blood work revealed thrombocytopenia. Posttransfusion thrombocytopenia was suspected. The patient was transfused with washed and leukoreduced platelets and treated with steroids and intravenous immunoglobulins. Laboratory studies demonstrated that she was homozygous for the HPA-Ib/1b platelet gene and positive antibodies against class 1 HLA and platelet glycoproteins. The patient responded well to treatment, with normalization of her platelet count.

## 1. Introduction

Posttransfusion purpura (PTP) is a rare complication of blood transfusion that occurs within 5–14 days after transfusion and results in a sudden severe thrombocytopenia which usually resolves spontaneously after two weeks. Recovery often occurs faster in patients treated with steroids, intravenous immunoglobulin (IVIG), or plasmapheresis. Implicated blood products include red blood cell component, platelet concentrate, and plasma component [1]. The antigen implicated in PTP is the human platelet antigen-1a (HPA-1a) [1]. Affected individuals are usually HPA 1a negative, and it is estimated that the prevalence is about 2% in the general population [1]. The disease has a striking female: male ratio of 26 : 1 which may be explained by the preexposure to blood product antigens through pregnancy or prior blood transfusion. Affected patients develop antibodies against these HPA-1a negative antigens during sensitization and destroy the host and donor platelets in subsequent transfusions. Diagnosis is made by identifying the presence of antibodies against the human platelet antigen in the patient's serum. Patients usually respond well to steroids, IVIG, or plasmapheresis. 

## 2. Case Presentation

A 43-year-old gravida 2 para 2 Caucasian female with a past medical history of anemia, uterine fibroids, and hyperthyroidism treated with radiation six years earlier presented to the emergency room with complaints of painless spontaneous gum bleeding and vaginal bleeding. There was no history of trauma or bleeding from any other site. The patient had no history of unusual or excessive bleeding, although she had a history of anemia secondary to prolonged menstrual bleeding from uterine fibroids, for which she previously received two units of packed red blood cells (PRBC). Her family history was noncontributory. 

Her blood pressure, heart rate, and respiratory rate were 118/65, 72, and 18, respectively. On examination, she was noted to have buccal purpura and scattered petechiae on her anterior abdominal wall and lower extremities. The rest of the exam was unremarkable.

Blood work revealed a hemoglobin (Hgb) of 9.2 g/dL (normal range—12.0–15.5), mean corpuscular volume (MCV) of 69.8 (normal range—80–100), platelets of 6,000 (normal range—150,000–450,000), and WBC of 9.2 k/mm cu (4.2–11.0). Three days prior to this presentation, her Hgb was 8.6 g/dL, MCV 68.3 fl, platelets of 330,000/uL, and WBC of 8.6 k/mm cu, following PRBC transfusion of two units for severe symptomatic anemia (Hgb 5.4) one week earlier. Further laboratory studies revealed a negative direct antiglobulin test and antinuclear antibody, normal levels of fibrinogen, prothrombin time, INR, D-dimer, and haptoglobin, with an elevated reticulocyte count. Peripheral blood smear showed no platelet clumping. The platelet morphology was normal, with markedly decreased number of platelets. The patient was started on intravenous methylprednisolone and IVIG. Repeat complete blood count showed a gradual trend towards an improvement in platelet count with normalization on day 7. Laboratory studies demonstrated a homozygous platelet genotype of HPA-Ib/1b with antibodies against HLA class I and platelet glycoproteins (GP IIb/IIIa and GPIa/IIa), which was consistent with PTP. This transfusion complication was reported to the blood bank.

## 3. Discussion

PTP is a rare complication of blood transfusion typically occurring 5–14 days after blood transfusion. PTP was first described in 1959 by van Loghem et al. [2]. Most have occurred in women sensitized during pregnancy [3]. However, prior blood transfusions and transplantation are associated with the development of PTP [4, 5].

The specific mechanism by which this destruction occurs is not clearly understood, but it is believed that the patient's platelets are destroyed when the antibody reacts with the antigen. HPA-1a is the most commonly implicated antigen in the PTP pathogenesis [1]. Interestingly to note that 1.5% of population is positive for HPA-1a, and one should expect a greater incidence of PTP [4, 5]. It is believed that certain human leukocyte antigen (HLA) genotypes such as HLA-B8, HLA-DRB3 ∗ 0101, and HLA-DQB1 ∗ 0201 are responsible for disease susceptibility [4].

 However, glycoprotein Ib/IIa, which is also known as human platelet antigen-5b (HPA-5b), has been reported to be responsible for PTP in two cases [6, 7]. Therefore, on a rare occasion other platelet antigens can be implicated in the pathogenesis of PTP [4–8].

Work up for other causes of thrombocytopenia is usually done, before a presumptive diagnosis of PTP can be made. Therefore, certain diseases should be excluded in patients presenting with otherwise unexplained thrombocytopenia. Chief among these is thrombotic thrombocytopenic purpura (TTP). In our particular case, it was very unlikely given the absence of fever, renal failure, a microangiopathic hemolytic anemia, or altered mental status. Disseminated intravascular coagulation (DIC) is another important part of the differential diagnosis. It is necessary to note that our patient had elevated fibrinogen levels and D-dimer. Moreover, her coagulation profile was normal, and no schistocytes were found in the peripheral smear. Heparin induced thrombocytopenia was ruled out because the patient did not receive any heparin products. Her only medication was oral iron, making drug-induced thrombocytopenia unlikely. Pseudothrombocytopenia was ruled out by the absence of platelet clumping on her peripheral smear. 

PTP has been shown to be an immunologic disorder with antibodies most commonly directed against the HPA-1a platelet antigen [8]. Interestingly to note that the recipient's own platelets (even if negative for implicated antigen) are destroyed. Several hypotheses exist, which explain this phenomenon: own platelets may absorb immune complexes, own platelets may acquire the implicated antigen from the antecedent blood transfusion, and autoantibodies to own platelets may be formed [4]. 

 The main modalities of treatment usually involve a combination IVIG, corticosteroids, or plasmapharesis. Consistent therapeutic success has been documented by starting with a high dose IVIG and corticosteroids. In our patient this approach resulted in the progressive rise of her platelets from 4,000 to 369,000. Plasmapharesis is usually a last resort. Some authors have tried starting therapy with steroids, and plasmapharesis with a resultant arrest of purpuric features, although the thrombocytopenia still persisted in these cases, resolving only after administration of IVIG [9]. However, it is important to note that IVIG on rare occasions is not clinically effective. Corticosteroids and/or plasmapheresis might be effective in such cases.

In addition, symptomatic treatment may occasionally be necessary with PRBC transfusion, depending on the severity of the patient's condition, since bleeding is a common feature of PTP. Platelet transfusion is not usually encouraged, as even HPA-1a negative platelets are also destroyed in this condition. Upon resolution of the acute event, affected patients are advised to receive transfusions only from HPA-1a negative donors. Blood bank must be notified in cases of PTP. Individuals who were diagnosed with PTP should receive only washed and/or HPA-1a negative blood products to decrease the future risk of PTP.
